# Antemortem and Postmortem Diagnosis of *Encephalitozoon cuniculi* in a Pet Rabbit (*Oryctolagus cuniculus*)—A Case Report

**DOI:** 10.3390/pathogens13121122

**Published:** 2024-12-19

**Authors:** Anca-Alexandra Doboși, Anamaria Ioana Paștiu, Lucia-Victoria Bel, Romelia Pop, Alexandru-Flaviu Tăbăran, Dana Liana Pusta

**Affiliations:** 1 Department of Genetics and Hereditary Diseases, Faculty of Veterinary Medicine, University of Agricultural Sciences and Veterinary Medicine Cluj-Napoca, 400372 Cluj-Napoca, Romania; anca.dobosi@student.usamvcluj.ro (A.-A.D.); dana.pusta@usamvcluj.ro (D.L.P.); 2 Department of General Surgery and ICU, New Companion Animals Veterinary Clinic, Faculty of Veterinary Medicine, University of Agricultural Sciences and Veterinary Medicine Cluj-Napoca, 400372 Cluj-Napoca, Romania; lucia.bel@usamvcluj.ro; 3 Department of Anatomic Pathology, Faculty of Veterinary Medicine, University of Agricultural Sciences and Veterinary Medicine Cluj-Napoca, 400372 Cluj-Napoca, Romania; romelia.pop@usamvcluj.ro (R.P.); alexandru.tabaran@usamvcluj.ro (A.-F.T.)

**Keywords:** *Encephalitozoon cuniculi*, encephalitozoonosis, rabbit, *Oryctolagus cuniculus*, case report, diagnosis

## Abstract

*Encephalitozoon* cuniculi infection in rabbits represents a true challenge in both diagnosis and treatment of the disease. This study aims to describe and analyze all methods of identifying the presence of the microsporidian in a rabbit through antemortem and postmortem methods. The patient manifested clinical signs of vestibular disease and mild renal symptoms with no significant improvement under treatment, which finally led to euthanasia. Serological and molecular tests confirmed positivity for *E. cuniculi* in serum and urine, feces, brain, kidney and urinary bladder, respectively. Histopathological findings showed suggestive inflammatory lesions of encephalitis and nephritis and no changes in the eye globe and liver, but with no identification of microsporidian spores. This is the first complete case report of *E. cuniculi* in a rabbit in Romania, as well as the first report of urinary bladder molecular testing with a positive result, which facilitates for further diagnosis exploration for rabbits.

## 1. Introduction

*Encephalitozoon cuniculi* is a microorganism from the phylum Microsporidia, with eukaryotic, unicellular, obligate intracellular, and spore-forming characteristics, that is currently considered to be a fungus [1,2]. The main host is the domestic rabbit, but its detection is present in many other species such as birds and even humans, hence why the disease is considered to be a zoonosis [3]. Human infection can occur in immunocompetent individuals, but the disease usually involves immunocompromised humans, children and elderly, as well as organ transplant recipients, clinically manifested with digestive and neurological symptoms [1,4]. Four different genotypes have been identified by analysis of the ITS region of the ribosomal genes as follows: Strain I was found predominantly in rabbits, strain II was the murine strain, strain III was found mainly in dogs, and, lastly, strain IV had prevalence in humans, dogs, and cats [2,3].

Transmission of *E. cuniculi* in rabbits has two main routes: horizontal and vertical. Horizontal transmission happens mainly through oral ingestion of food and water contaminated with excretions and rarely through inhalation of spores [4]. The intrauterine mode of transmission represents the vertical route, from doe to kit, which has mainly been demonstrated to occur through spore transmission in the ocular structures, more exactly the eye lens, as well as in the brain, liver, and kidney tissues of the offspring [5]. Host immunity involves cell-mediated immunity, which is the superior one that occurs with the help of CD4^+^, CD8^+^ T lymphocytes and cytokines, and humoral immunity through the Immunoglobulin M (IgM) and Immunoglobulin G (IgG) antibody production in response to the organisms’ infection [4,6].

Diagnosis of encephalitozoonosis in rabbits begins by assessing the clinical status, where the acute manifestation shows vestibular signs as a result of central nervous system (CNS) lesions, such as head tilt, ataxia, nystagmus, paresis or hemiparesis, tremors, and seizures and rolling, together with urinary incontinence. Damage to the renal system can also show unspecific signs of anorexia, polyuria, and polydipsia, accompanied or not by cystitis, while eye globe lesions usually include unilateral phacoclastic uveitis, secondary glaucoma, and cataracts [2]. Nevertheless, subclinical disease is the most common case of infection in rabbits, which is the reason why the sole clinical examination is not sufficient for encephalitozoonosis confirmation, and other types of diagnosis methods need to be considered. These methods include antemortem *E. cuniculi* detection, such as serological diagnosis, paraclinical blood and imaging tests, and molecular identification of the microorganism in the urine, feces, and cerebrospinal fluid (CSF), as well as post-mortem techniques, which include histopathological and molecular testing of the targeted organs [4,7,8].

Treatment of encephalitozoonosis in rabbits remains a real challenge as it cannot be entirely cured, and acute disease is usually attempted to be managed by reducing spore-mediated inflammation and spore proliferation, together with management of neurological signs and supportive therapy. Main medication includes an anti-parasitic protocol with fenbendazole at 20 mg/kg orally q24h for 28 days, steroidal or non-steroidal systemic anti-inflammatories, systemic antibiotherapy for managing concurrent secondary infections, and topical medication for improvement of ocular signs [4,7]. Due to the situation, preventing *E. cuniculi* spread from one rabbit to the other, and even to humans, is very important for disease control. This can be achieved by periodical serological testing in large rabbit populations, resulting in the elimination of positive individuals [4,9] and the obtainment of an encephalitozoonosis-free rabbitry, preventive administration of fenbendazole, especially for rabbits newly introduced in a population [7], and thorough disinfection of the rabbits’ environment, with limitations of their contact with other animals or wildlife that possess a risk of pathogen transmission [4].

The aim of this study is to describe a confirmed case of encephalitozoonosis in a pet rabbit, starting with its clinical presentation and medical management, the various diagnosis methods used for *E. cuniculi* identification, and the comparison between these, thus offering a complete picture of how this pathogen can affect a rabbit’s organism and an analysis of what can be done to improve future occurrences.

## 2. Materials and Methods

### 2.1. Patient General Data

A 5-year-old male, neutered, vaccinated against myxomatosis and rabbit hemmhoragic disease strain 1 (RHDV1), and of mixed breed, was presented to the Clinic of New Companion Animals of the Faculty of Veterinary Medicine of Cluj–Napoca, Romania, as an emergency, with complaints of lethargy, inappetence, reduced fecal output, and presence of neurologic signs with impossibility of keeping its balance. On clinical evaluation, the patient weighed 3.1 kg, had body condition score (BCS) of 4/5, presented hypothermia (36.5 °C), had a moderate head tilt to the left side, ataxia, and a distended abdomen with dense content on palpation and no gut sounds present on auscultation. The animal was hospitalized for a total of 13 days when it was euthanized.

All diagnosis methods and treatment were performed with the written owner’s consent and with approval by the Animal Ethics and Welfare Committee of the University of Agricultural Sciences and Veterinary Medicine, Cluj–Napoca, Romania (No. 320/3 June 2022).

### 2.2. Therapeutic Management

The animal was hospitalized, and intensive care was instituted with fluid therapy (Ringer^®^ solution with a lidocaine and ketamine CRI) 10 mL/kg/h CRI, Buprenorphine 0.03 mg/kg s.c. q8h, Metoclopramide 0.5 mg/kg s.c. q12h, and housing in an incubator at 30 °C for improvement of body temperature. Subsequently, additional therapy included fluid therapy (Ringer^®^ solution mixed with pyridoxine hydrochloride, DL-Aspartic acid and acetylcysteine) 10 mL/kg/h CRI, levetiracetam 20 mg/kg orally q12h, trimethoprim/sulfametoxazole 30 mg/kg orally q12h, and assisted feeding with EmerAid© Intensive Care Herbivore 10 mL/kg q4-6h, depending on the patient’s appetite. For sedation, in order to perform paraclinical tests or during seizures, Midazolam 0.5 mg/kg i.m. or i.v. was used. Fenbendazole, the usual anti-parasitic medication used in the treatment of encephalitozoonosis, was not administered in this case due to the hepatic damage that this drug could further induce, as well as the late confirmation of *E. cuniculi* infection through serological diagnosis.

### 2.3. Paraclinical Tests During Hospitalization

During the course of the rabbit’s hospitalization, multiple tests were recommended to the owner and were performed according to financial resources and patient’s evolution. On Day 1 of hospitalization, thoracic and abdominal X-rays were performed under sedation of the patient.

Feces eliminated by the patient in the first 2 days of hospitalization were collected and sent for coproparasitological examination. This was conducted through the centrifugal flotation method using concentrated zinc sulphate solution (1.18 g/mL) and flotation method using saturated sodium chloride solution (1.2 g/mL).

Biochemical blood analysis was performed on Day 1 and Day 7 of hospitalization from blood collected from the lateral saphenous vein using a 23 G needle and a heparinized blood collection tube, followed by blood analysis using the VetScan VS2 Chemistry Analyzer (Abaxis, Union City, CA, USA).

### 2.4. Serological Diagnosis of E. cuniculi

Due to owner’s financial constraints, serological testing for *E. cuniculi* diagnosis was only performed on Day 10 of the rabbit’s hospitalization. Approximately 2 mL of blood were drawn using the lateral saphenous vein with a 23 G needle and a clot-activator blood-collection tube. The sample was centrifuged at 5000 rpm for 10 min and sera was collected for testing. Further, a commercial indirect enzyme-linked immunosorbent assay (ELISA, Medicago^®^, Uppsala, Sweden) was used for qualitative anti-*E. cuniculi* antibody detection using the manufacturer’s instructions. The tested results can be interpreted as positive or negative.

Additionally, serum was also sent to the clinical laboratory LABOKLIN (Bad Kissingen, Germany) for quantitative IgM and IgG titer measurement through immunofluorescent antibody detection (IFAT). The cut-off of the fluorescent test was 1:80.

### 2.5. Molecular Genetics Diagnosis of E. cuniculi

#### 2.5.1. DNA Extraction

DNA extraction has been conducted from samples of urine and feces collected antemortem and brain, kidney, eye lens, urinary bladder, liver, lungs, and heart collected postmortem using different extraction kits.

Urine, collected by manual compression of the urinary bladder, was later centrifuged at 5000 rpm for 5 min, and supernatant was removed. DNA extraction was done using the Maxwell^®^ RSC Genomic DNA Kit (Promega, Madison, WI, USA) following the manufacturer’s protocol for cell pellet samples.

Fecal pellets were collected from the patient’s cage during the first days of hospitalization, when fecal output started improving. DNA extraction was done using the QIAamp^®^ Fast DNA Stool Mini Kit (Qiagen, Hilden, Germany), following the manufacturer’s instructions.

Tissue collection was conducted during necropsy, with small sections from each organ being stored in 2 mL Eppendorf tubes and stored at −18 °C. DNA extraction was later done using the ReliaPrep™ gDNA Tissue Miniprep System (Promega, Madison, WI, USA) following the manufacturer’s protocol.

#### 2.5.2. PCR Analysis

Real-time PCR, also known as quantitative PCR (qPCR), has been the method of choice for DNA amplification of urine, feces, and tissue samples of the brain, kidney, eye lens, urinary bladder, liver, lungs, and heart. The Encephalitozoon cuniculi Genesig^®^ Standard Kit (Primerdesign Ltd.^TM^, Chandler’s Ford, Hampshire, UK) was used by the manufacturer’s protocol, with 5 µL of DNA added to each of the well prepared. The qPCR was run in one cycle at 95 °C for 2 min, followed by 50 cycles at 95 °C for 10 s and 60 °C for 1 min, and, lastly, one cycle at 60 °C for 1 min. The Azure Cielo^TM^ Real-Time PCR System (Azure Biosystems, Dublin, CA, USA) was utilized for PCR amplifications. Positive control (provided by the Encephalitozoon cuniculi Genesig^®^ Standard Kit) and negative control (distilled ultrapure water) were included in each run.

### 2.6. Necropsy and Histopathological Diagnosis of E. cuniculi

Necropsy was performed after euthanasia of the rabbit. During necropsy, brain, eye, kidney, and liver tissues were collected and initially preserved in 10% neutral buffered formalin. After a 48 h fixation period, samples were trimmed and placed in fresh 10% neutral buffered formalin. The tissues then underwent dehydration through a graded series of ethyl alcohol baths, cleared in xylene, and embedded in paraffin at 58 °C for 5 h using low-melting-point paraffin. Sections 2 µm thick were cut from the paraffin blocks using a rotary microtome and were stained by hematoxylin and eosin (H&E) [10]. Permount and coverslips were applied to the slides, which were examined under an Olympus BX51 microscope (objectives 100× and 200×), with imaging performed using an Olympus SP350 camera and cellSens Software Standard Version 4.2 CS-ST-V4.2.

## 3. Results

### 3.1. Patient Clinical Evolution

**Figure 1:**
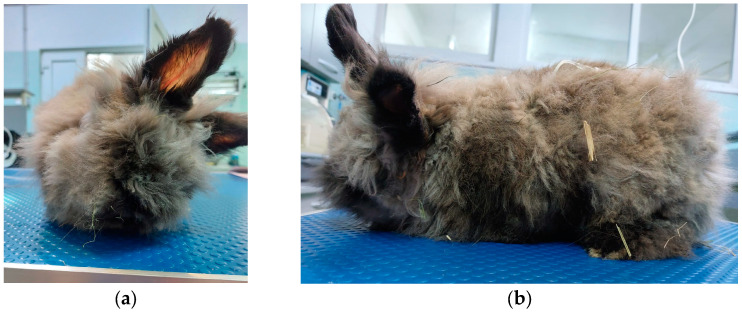
Left head tilt of the rabbit: (**a**) cranial view; (**b**) left lateral view.

During the course of 13 days of hospitalization, the rabbit’s general state initially improved by the presence of fecal output, moderate appetite, and a better general state, but it continued to present a head tilt to the left (Figure 1) and ataxia, together with newly observed symptoms like polydypsia and polyuria. Based on clinical manifestation, the main two differential diagnoses included encephalitozoonosis and otitis media/interna. The patient’s state was stagnant, with the symptoms described before for approximately 10 days of hospitalization. Unfortunately, from Day 11, the rabbit’s neurological symptoms worsened, with a more severe head tilt to the left and hypersensitivity to handling, followed by the appearance of seizures with rolling and urinary incontinence. Euthanasia was recommended to the owner and was performed after 13 days of hospitalization.

### 3.2. Paraclinical Tests During Hospitalization

The X-rays results revealed a distended stomach with moderately radio-opaque content and intestines with areas of radio-opaque and radio-lucent content, together with mild pulmonary densification (Figure S1). No other relevant changes were observed.

Coproparasitological examination revealed a negative result.

Biochemical blood analysis results are presented in Table 1. These showed signs of dehydration and liver impairment [hypoalbuminemia, hyperglobulinemia, low values of blood urea nitrogen (BUN) and creatinine, and high value of alkaline phosphatase (ALP)], as well as slight renal impairment (high values of calcium and potassium, low value of phosphorus, and imbalance of Ca:P ratio). Additionally, on Day 7, the blood glucose level was high, but the parameter values remained more or less similar for both analyses. Based on these results, hepatic encephalopathy was added to the list of differential diagnoses.

### 3.3. Serological Diagnosis of E. cuniculi

Qualitative anti-*E. cuniculi* antibody detection by ELISA revealed a positive result. The following quantitative detection of antibody titers (IFAT) resulted in an IgM titer of 1:320 and an IgG titer of 1:320. Therefore, both antibody measurements were considered positive.

### 3.4. Molecular Genetics Diagnosis of E. cuniculi

Real-Time PCR revealed positive results for urine, feces, brain, kidney, and urinary bladder, while the eye lens, liver, lungs, and heart turned out all negative for specific *E. cuniculi* DNA.

### 3.5. Necropsy and Histopathological Diagnosis of E. cuniculi

**Figure 2:**
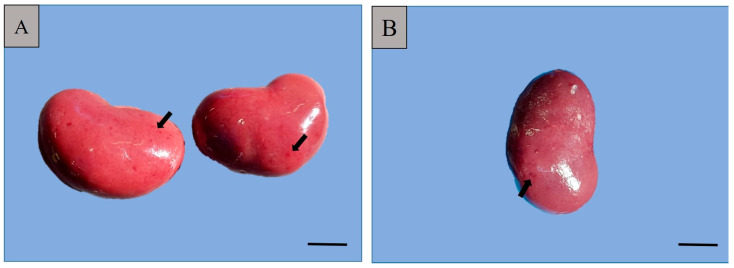
Macroscopical aspect of kidneys examined, 1 cm scale: (**A**) left and right kidneys showing subcapsular petechiae (black arrows); (**B**) the right kidney, presence of subcapsular petechiae (black arrow).

Macroscopical findings observed during necropsy were represented by the presence of bilateral subcapsular petechiae (Figure 2).

**Figure 3:**
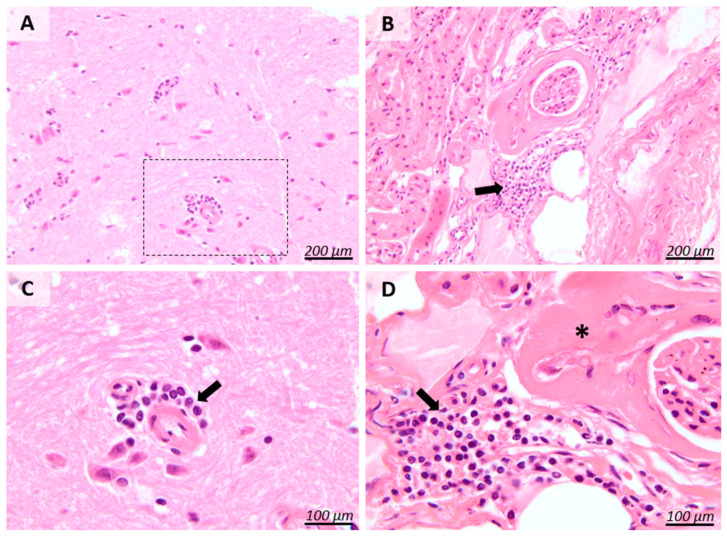
Histopathological images of the cerebrum (Images (**A**,**C**)) and kidneys (Images (**B**,**D**)). Cerebrum: multifocally, the perivascular Virchow–Robin spaces are distended and infiltrated by a moderate number of lymphocytes and plasma cells (Image (**C**), black arrow). Kidneys: the renal corpuscle capsule is markedly thickened by fibrous connective tissue (Image (**D**), asterisk), and multifocally, the renal interstitium is infiltrated by a moderate number of lymphocytes and plasma cells (Image (**D**), black arrow). H&E stain.

Histological analysis of tissues revealed unspecific lesions of both encephalitis and nephritis, with mainly the presence of inflammatory infiltrate of lymphocytes and plasma cells (Figure 3), while eye globe and liver showed no pathological changes.

## 4. Discussion

Diagnosing *E. cuniculi* in rabbits through various methods and finding the most accurate options remain some of the main objectives of research in this field. Determining anti-*E. cuniculi* antibodies remains one of the standard techniques for diagnosing the microsporidia in a rabbit antemortem, where identification and/or measurement of IgM and IgG can be conducted. Depending on the phase of the infection, it has been reported that a high titer of IgM is indicative of an early or acute infection and a high titer of IgG is found in a chronic or latent infection, while both IgM and IgG simultaneously elevated suggests the presence of an active infection, whether it is an acute, a reactivated infection, or a reinfection [12]. The serological methods that have been previously used for this diagnosis are enzyme-linked immunosorbent assay (ELISA), indirect fluorescent antibody test (IFAT), carbon immunoassay (CIA), Western blot analysis, and C-reactive protein (CRP) measurement, depending on availability and sensibility [13]. In this study, qualitative ELISA was performed for antibody identification, followed by quantitative titer measurement of both IgM and IgG through IFAT, with both tests having a positive result. ELISA has been the method of choice in several other studies on diagnosing *E. cuniculi* in rabbits [14,15,16,17] due to its high sensibility and method standardization, such as qualitative and quantitative testing options. IFAT, the other technique that can be used to determine the levels of IgM and IgG [18,19], has also shown promising results, with significant positive correlations having been found between ELISA and IFAT [17]. Using IFAT, there were high titers of both IgM and IgG obtained, 1:320, respectively, which indicates that the infection was active in the studied rabbit, but with no clue to the exact moment of the rabbit’s contact with the microsporidian. The drawback noticed by the authors is the larger number of samples necessary to have the ELISA test done using the commercial kit in comparison to the IFAT method, which can be conducted on single individuals by using antigen-coated slides. CIA is another method that comes with a relatively low-cost, easy, and fast technique, but that can only detect IgG antibodies [20]. Significant correlations have also been found between ELISA and CIA methods, as well as between CIA and IFAT [17,20]. The other two techniques mentioned before, Western blot analysis and CRP measurement, have a rather less frequent use due to labor-intensive features and non-specificity to encephalitozoonosis, respectively. Due to the wide application of serology in *E. cuniculi* diagnosis, although with its limitations, it continues to be the most useful diagnostic tool, both for large populations and also for single rabbits.

Identification of specific *E. cuniculi* DNA through molecular genetics techniques from various biological materials represents another method that is increasingly performed among rabbits. The tissues that have been previously tested with these types of methods are represented by the brain, kidney, liver, lungs, heart, spleen, and intestines, sampled solely postmortem, and ocular globe or eye lens, which can also be acquired antemortem by enucleation/phacoemulsification [18,21]. Other types of materials reported for molecular analysis are urine, feces, and cerebrospinal fluid (CSF), but with a relatively low sensibility and prevalence among most studies [18,22,23]. The main molecular methods performed for this diagnosis are conventional PCR, nested PCR, and real-time PCR, with different results according to the authors. The conventional PCR seems to have the most limited application, where the positivity is reported to be low [18,23,24] in tissues, urine, and feces, while the prevalence is higher from ocular globes that carry a higher load of spores through intrauterine transmission [5,18]. Nested PCR presents a wider use with better results because this method relies on the succession of two PCR reactions using different primer sets in which the product of the first reaction is amplified again in the second one [18]. For this study, real-time PCR or qPCR was performed for all materials tested, with this method being chosen for its advantages of increased throughput, a lower risk of contamination from PCR amplicon carry-over, and the higher sensibility for quantitative DNA detection [25]. Positivity was obtained for the brain and kidney, which coincides with other studies where these organs are the most prevalent for *E. cuniculi* DNA identification [18,21] since the rabbit manifested the acute form of the disease with neurological and renal symptoms. An interesting finding was the positivity of the urinary bladder, which had never been previously reported to have been tested. This must be in accordance with the positivity also shown by the urine, assuming that residual urine spores remained on the urinary bladder mucous membrane. qPCR positivity was also shown for the feces sample, also explained by the acute moment of spore shedding during the infection. Eye lens, liver, lungs, and heart provided a negative molecular result, similar to other reports [18,21] where lower prevalences were found in these organs due to *E. cuniculi*’s higher predilection for brain and kidney localization. What is important to emphasize are the limitations represented by the materials tested, such as a low or non-uniform spore load in the organ section tested, as well as the moment of infection when the materials have been sampled, since *E. cuniculi* has a different distribution in the organs according to the moment of infection and intermittent shedding through excretions occurs [1,22]. Continuous effort is put into the improvement of these methods for *E. cuniculi* diagnosis. However, postmortem testing of predilection organ tissues has proved to have the highest sensibility for PCR techniques so far.

Histopathological diagnosis of *E. cuniculi* has been the last method applied for this case, where the brain, kidney, eye globe, and liver of the rabbit have been examined. Inflammatory lesions of lymphoplasmacytic encephalitis and nephritis, respectively, accompanied by fibrosis in the renal tissue, were identified using the H&E stain. Even if these lesions in a rabbit are suggestive of an *E. cuniculi* infection, it must be noted that they are not pathognomonic. Other reports on larger rabbit groups with prior encephalitozoonosis clinical signs have identified similar lesions of granulomatous meningoencephalitis and chronic interstitial nephritis, the brain and kidney being the main organs of predilection [4]. These types of lesions are described by the presence of perivascular cuffs composed of plasma cells, lymphocytes, and rarely macrophages in the cerebral tissue [26], while the kidneys present interstitial lymphocytic proliferation and necrosis of tubular epithelium, accompanied by fibrosis associated with scar-like tissue in more severe cases [2,26,27]. Although the eye globe is usually the third-most prevalent organ in *E. cuniculi* infections in rabbits, no changes were observed in this study since the patient showed no ocular symptoms. Histological eye globe changes can usually be observed in rabbits showing signs of phacoclastic uveitis or cataract, where the lens capsule is ruptured or thinned, and lens epithelial cells are degenerated or disorganized [28]. Less frequently, the liver can be affected with lesions of mild hepatocyte degeneration and mononuclear cellular infiltrates [27], but no changes were observed in this study. An important finding is related to the identification of spores in all tissues mentioned, either in a mature stage or arranged in cyst-like aggregates, which can occasionally be found inside parasitophorous vacuoles intracellularly, as well as in the extracellular space [2,26]. However, this histological discovery relies in most cases on stains such as Gram stain, Periodic Acid–Schiff stain, Ziehl Neelsen stain, and modified trichrome stains, which proved to be more sensitive to spore detection than H&E stain alone [4,26]. Even so, the display of tissue sections with a low or absent spore load for histological examination represents a limitation that could be applied to this study. Although severe histological lesions suggestive of encephalitozoonosis can be found in rabbits, it has been shown that they do not necessarily correlate with the manifestation of clinical symptoms, which implies that the severity of the histological lesions cannot establish *E. cuniculi* as the cause of disease [8].

Additional routine paraclinical tests, which have been performed in this case as well, are useful in regard to assessing the patient’s clinical state and excluding other differential diagnostics. Complete blood count (CBC), biochemical blood analysis, serum protein electrophoresis, and imaging investigations are some of the options considered in an acute manifested encephalitozoonosis. In this study, thoraco-abdominal X-rays were performed, revealing unspecific changes in accordance with gastrointestinal syndrome. Advanced imaging techniques such as computerized tomography (CT) or magnetic resonance imaging (MRI) would be better options to help exclude differential diagnostics of vestibular clinical signs, like otitis media/interna [29]. Unfortunately, the CT scan was refused in this case due to the owner’s financial constraints. On the other hand, blood biochemical tests showed low values of albumin, BUN, creatinine, and phosphorus, as well as high values of globulin, ALP, calcium, potassium, and glucose. CBC was not performed for this rabbit, again due to financial constraints of the owner. According to another report [7], rabbits serologically diagnosed with *E. cuniculi*-manifesting neurological signs can often have higher serum urea and lower phosphorus levels, while the ones with renal signs present a lower packed cell volume, increased heterophil count, and lower phosphorus and potassium values. Anemia was also noted in a recent study [30], associating it with rabbits infected with encephalitozoonosis, where the inflammation and the renal damage present in the disease could lead to an impaired erythropoietin production that explains this hematological change. Contrary to other studies, where BUN and creatinine tend to be elevated in an *E. cuniculi* infection [31,32] due to renal insufficiency, this case reported values below the reference range, which could indicate a hepatic insufficiency or muscle mass loss [31]. Varied results, such as elevated ALP, cholesterol, phosphorus, and glucose, have been observed in seropositive rabbits before [32]. Hyperglycemia in a rabbit can often be associated with stress or concurrent GI disorders, which is why it cannot be named characteristic of encephalitozoonosis [30]. Lastly, the initial elevated globulin level of the patient is similar to other studies, where serum protein electrophoresis revealed a high γ-globulin fraction and a low albumin-to-globulin ratio in clinically ill rabbits with suspected *E. cuniculi* infection [7,33]. Although the obtained blood results are compliant with other studies, it must be remembered that all of these do not follow a characteristic pattern in the disease, and they must be always interpreted in a clinical context.

Although the current study’s main focus was on the multiple diagnosis methods used, therapy and prophylaxis of encephalitozoonosis in rabbits are important aspects to be discussed. The patient in question received mainly supportive therapy, managing pain, dehydration, and neurological symptoms in accordance with the paraclinical tests done at the time and the animal’s clinical evolution. Even if encephalitozoonosis was high on the differential diagnosis list, fenbendazole treatment was not administered due to the concern of further hepatic damage that this could cause, as well as the late confirmation of the disease by serological diagnosis. One study demonstrated that fenbendazole in rabbits proved to be more efficient when administered before the infection as a prophylactic agent for a course of 7 days at 20 mg/kg orally daily, rather than as a treatment post-infection, for the 28-day course in the same dosage [34]. This emphasizes that the outcome for our case would have most likely been the same even with fenbendazole administration, considering the severity of clinical symptoms and the lower efficacy in an acute disease. In terms of reducing inflammation caused by cell-host rupture, our patient did not receive any steroidal anti-inflammatory therapy, such as dexamethasone, which mainly proved to have an immunosuppressive effect and not a therapeutic one [4,7], nor non-steroidal anti-inflammatories, where the further potential renal damage also caused by *E. cuniculi* could present a risk [4]. The use of antiemetic drugs, such as metoclopramide, and anticonvulsants, such as diazepam or midazolam, have previously been reported and are in accordance with our treatment choice [7]. Levetiracetam, an antiepileptic agent, is a rather efficient option in most species, but further studies on rabbits are required. Broad-spectrum systemic antibiotherapy with trimethoprim-sulfamethoxazole, enrofloxacin, or oxytetracycline has been documented for treating concurrent primary or secondary bacterial infections, which we also took into consideration, but its efficacy remains to be further tested in such cases. Considering that therapeutic options for the acute manifestation of encephalitozoonosis can be unrewarding, and that the prognosis is, most times, unfavorable, prophylactic measures remain the method of choice for reducing the spread of *E. cuniculi*. For a pet rabbit that is housed indoors and with no contact with other animals, the risk of horizontal transmission with the pathogen is relatively low. However, the risk of vertical transmission from their mother at birth is still in question, which is why periodical serological testing is recommended even in isolated animals, but even more so in larger rabbit populations [7]. Assuring a stable and stress-free environment, as well as prophylactic administration of fenbendazole to the rabbit and thorough disinfection of the animal’s enclosure, are also measures recommended to prevent *E. cuniculi* infection [1], not only from one rabbit to another but also from a rabbit to their owner, as the disease is classified as a zoonosis with a potential threat to immunocompromised humans.

## 5. Conclusions

Encephalitozoonosis is a highly prevalent disease in rabbits, where the acute form can unfortunately cause increased mortality. This case describes the confirmation of *E. cuniculi* infection mainly through serological and molecular genetic diagnosis, but the clinical presentation and histopathological findings could also indicate the cause of the disease being this microsporidian. Although numerous studies on large rabbit populations have been previously conducted on the diagnosis methods of *E. cuniculi*, this is the first documented case report in Romania and the first attempt at testing the urinary bladder through molecular genetic techniques that revealed a positive result. Further studies on bigger rabbit groups exploring these diagnosis methods and their statistical comparison are expected to occur in the country.

## Figures and Tables

**Table 1:** Biochemical blood analysis of the patient 1 week apart.

Parameter	Day 1	Day 7	Reference Range [11]
ALB (g/dL)	2.3	2.3	2.8–4
ALP (U/L)	65	44	6–14
ALT (U/L)	46	28	52–80
Amylase (U/L)	204	135	82–343
Total Bilirubin (mg/dL)	<0.1	0.3	0.1–0.5
BUN (mg/dL)	4	9	
Calcium (mg/dL)	14.2	13.4	7.6–12.2
Phosphorus (mg/dL)	2.5	1.8	3–6.2
Creatinine (mg/dL)	0.7	1.1	1–2.2
Glucose (mg/dL)	150	281	109–161
Sodium (mEq/L)	142	135	138–148
Potassium (mEq/L)	6.0	5.1	3.4–5.1
Total Protein (g/dL)	7.2	5.6	6.1–7.5
GLOB (g/dL)	4.9	3.3	2.1–3.7

## Data Availability

The data presented in this study are available on request from the corresponding author due to owner privacy reasons.

## Supplementary Materials

The following supporting information can be downloaded at: https://www.mdpi.com/article/10.3390/pathogens13121122/s1, Figure S1: Thoracic and abdominal X-rays: (S1) right latero-lateral view; (S2) left latero-lateral view; (S3) dorso-ventral view.
