# Supplementary material for: Antemortem and Postmortem Diagnosis of Encephalitozoon cuniculi in a Pet Rabbit (Oryctolagus cuniculus)—A Case Report
Source: Pathogens. 2024 Dec 19;13(12):1122. doi: 10.3390/pathogens13121122 (PMC11676482; doi:10.3390/pathogens13121122)
Supplement: Supplementary file 1 [file pathogens-13-01122-s001.zip › pathogens-3350259-supplementary.pdf]

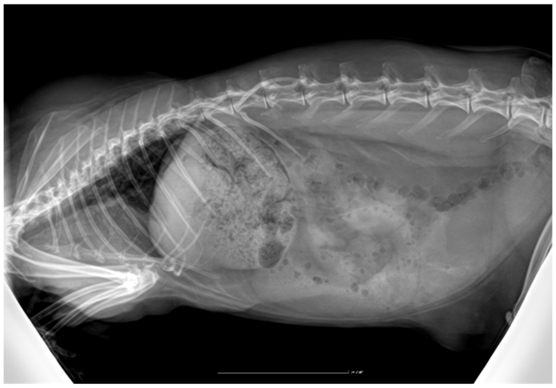

S1

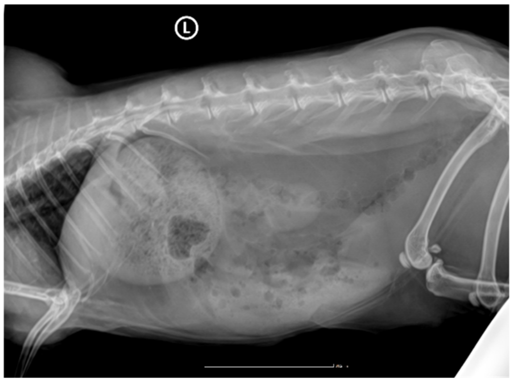

S2

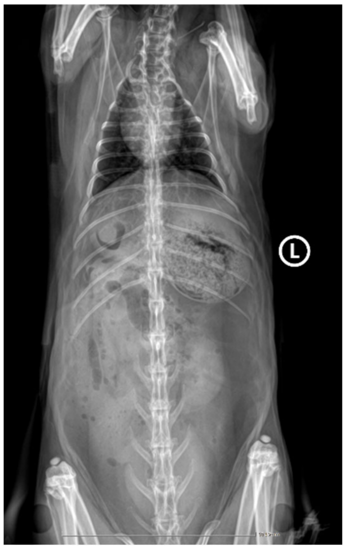

S3

Figure S1: Thoracic and abdominal X-rays: (S1) right latero-lateral view; (S2) left latero-lateral view; (S3) dorso-ventral view.
